# "Sparks that became a little light over time": A qualitative investigation of musicking as a means of coping in adults with PTSD

**DOI:** 10.1371/journal.pone.0228050

**Published:** 2020-01-30

**Authors:** Iftach Ophir, Rebecca Jacoby

**Affiliations:** 1 Medical Psychology Graduate Program, School of Behavioral Sciences, Academic College of Tel Aviv-Yaffo, Tel Aviv-Yaffo, Israel; 2 Medical Psychology Graduate Program, Stress, Hope and Cope Lab, School of Behavioral Sciences, Academic College of Tel Aviv-Yaffo, Tel Aviv-Yaffo, Israel; Anadolu University, TURKEY

## Abstract

This article investigates the experience of musicking (the performance of musical activity) among people coping with post-traumatic stress disorder (PTSD). Using qualitative research methods, we conducted semi-structured in-depth interviews with 10 male participants in a music project for people coping with PTSD induced by war and terrorism. The project consists of individual music lessons once a week and a musical enrichment group that meets once a month. Group meetings include workshops and lectures by music professionals and artists, during which participants are exposed to diverse musical content. Following an interpretive phenomenological content analysis, we were able to identify two central themes arising from the interviews: musicking as an intra-subjective experience and musicking as mediator of inter-subjective relationships. A further analysis revealed three superordinate themes: musicking as a secure place, musicking as a dialectic experience, and musicking as a means for identity reconstruction (bridging between past, present, and future). From this we concluded that for our interviewees musicking is a secure place for their wounded self, which allows the reconstruction of a coherent personal narrative while conducting a dialectic encounter with the trauma and its symptoms via nonverbal language. Consequently, we recommend musicking as a therapeutic tool for people coping with war-induced PTSD from both intrapersonal and interpersonal perspectives.

## Introduction

### Post-Traumatic Stress Disorder (PTSD)

PTSD may develop after a stressful event or situation (short-term or ongoing) of an exceptionally threatening or catastrophic nature, which is likely to cause pervasive distress in almost anyone [1]. The symptoms of the disorder can be divided into three categories. The first includes invasiveness and re-experiencing the trauma in the form of sudden flashbacks, dreams, thoughts, and emotional distress when exposed to stimuli that evoke the traumatic experience. The second is characterized by an avoidance of proximity to people, places, activities, and thoughts which may evoke the initial traumatic experience and also includes substance abuse. The third category, hyperarousal, is expressed in tension, sleep disorders, difficulty in concentrating, and sudden uncontrollable outbursts of anger. Symptoms may appear for a limited time or be continuous. PTSD is diagnosed when symptoms persist beyond one month [2]. The prevalence of PTSD in the general population is between 0.5%–3.5%, depending on cultural, genetic, and demographic factors. PTSD often appears in comorbidity with other psychiatric disorders such as depression, anxiety, and somatic disorders [3].

A number of theoretical approaches have attempted to explain the PTSD etiology. Verhaeghe and Vanheule [4] offered a psychoanalytic perspective on the development of PTSD, regarding trauma as the result of confrontation with an identity threatening incident that cannot be elaborated by the victim in a normal, associative, and meaningful way. Thus, PTSD can be related to a preexisting, actual neurotic structure that has developed due to lack of parental sensitivity in childhood.

Behavioral theories use "classical conditioning" to explain PTSD, while cognitive theories emphasize the difficulty in processing a traumatic experience that does not correspond to existing schemes [5]. According to the dual representation theory of PTSD, there are two separate presentations of memory: verbal and situational. Both include sensory, emotional, and physiological information regarding the event. However, while verbal memory is accessible to the individual and therefore the information it contains can be verbally described, situational memory is not verbally accessible, so that certain stimuli connected to the trauma can evoke traumatic experiences stored within it [6].

Rothschild [7–8] used a neurological perspective to argue that in PTSD the traumatic experience is retained in an implicit “somatic memory” focused on sensory and motor aspects rather than being verbal and explicit (declarative memory). Her findings indicated that the limbic system, which includes the amygdala and hippocampus, is the focus of PTSD pathology. In response to a traumatic event, the amygdala is hyperstimulated, causing suppressed activity in the hippocampus, which is involved in encoding the event to memory. Rothschild [7] adopted the model described by Levin [9], who argued that events are normally encoded in memory, both implicitly and declaratively, via five components: visual, sensorial, behavioral, emotional, and semantic. When the hippocampus is suppressed, one (or more) of these five components is not encoded in memory, causing the event to be encoded in memory in a dissociated manner.

Research in the field of neuroscience and neurobiology has attempted to provide a deep description of the neural abnormalities underlying PTSD. Based on neuroimaging findings, functional neuroanatomical models of PTSD have been shaped by findings of decreased activation of the medial prefrontal cortex (mPFC) and increased activation of the amygdala in response to trauma- or fear-related stimuli [10]. Admon, Milad and Hendler [11] offered a theoretical model suggesting that the amygdala and dorsal anterior cingulate cortex represent predisposing risk factors for developing PTSD, whereas dysfunctional hippocampal-ventromedial prefrontal cortex (vmPFC) interactions may become evident only after developing the disorder. Recent research in neuroscience has focused on identifying neural networks that underlie PTSD mechanism and symptoms [12, 13]. Three intrinsic connectivity networks in the brain–the central executive, salience, and default mode networks–have been identified as crucial to psychopathology including PTSD. Each network has been associated with specific clinical symptoms observed in PTSD: cognitive dysfunction (central executive network), increased and decreased arousal (salience network), and an altered sense of self (default mode network) [12]. Moreover, two brain networks have been found in which weaker connectivity of the hippocampus and cortex regions was linked to higher PTSD re-experiencing symptoms. Conclusions based on these finding have suggested that PTSD re-experiencing symptoms are linked to weakened connectivity in a network involved in providing contextual information [13].

Although the various theories have described PTSD mechanisms in different ways, there is a consensus that PTSD is an outcome of a process in which traumatic memories are encoded emotionally, cognitively, and perceptively and are dissociated from other memories. These memories are encoded in a scattered and incoherent manner, thus making it difficult to describe the traumatic events as part of a personal narrative [14]. The inability to symbolically process traumatic memory (either verbally or in any other symbolic way) is the core of the mechanism of PTSD [15].

### Treating PTSD

Chronic PTSD is especially resistant to treatment, apparently because extreme trauma significantly affects the brain [16]. A number of treatments have been attempted including pharmacological, cognitive, behavioral, and creative art therapies. Medication includes anti-anxiety drugs and antidepressants such as SNRI/SSRI and Mitrazapine. In rare cases antipsychotics are also used [17]. In recent years, findings have pointed to the efficacy of cannabinoids for controlling nightmares [18]. Further studies have attested to the efficacy of using MDMA (Methylenedioxymethamphetamine) during the course of psychotherapy with patients coping with PTSD [19].

Psychological approaches, such as trauma-focused psychotherapy that relies on exposure techniques and cognitive behavioral therapy (CBT), have been found effective in treating PTSD [20]. The underlying theoretical assumption of CBT is that the anxious person possesses a certain cognitive scheme which should be activated while alternative schemes are suggested [21]. Prolonged Exposure (PE), which is based on processes of habituation, extinction, and emotional regulation, has been found highly effective [22]. EMDR (eye movement desensitization and reprocessing) has also become prevalent in recent years. According to supporters of EMDR, a bilateral activity such as eye movement enables a reprocessing of the trauma evoked from memory [23]. Narrative PTSD therapies enable the construction of a coherent personal narrative which includes the traumatic event [24]. Traumatic memories can thus be symbolically organized, reconstructed, and processed [15].

The therapeutic methods described above mostly rely on verbal discourse to evoke memories and process the contents of the trauma. However, these methods are not always successful in addressing the content of the trauma as encoded in nonverbal memory [25]. Sarid and Huss [26] suggested instead that nonverbal therapies allow the person access to the trauma via multiple sensory channels.

In short, while PTSD is becoming more prevalent, theories to describe the mechanisms underlying the phenomenon and its various treatments are still under dispute. Findings indicating the difficulty of processing the traumatic memory verbally have encouraged us to investigate the experience of a nonverbal mode of expression–musicking–among veterans coping with PTSD.

### Music and musicking as therapeutic tools

Most people can perceive music, tones, timbre, pitch, intervals, melodic contours, harmony, and rhythm. However, listening to music is not just auditory; it also involves emotion and movement [27]. The relationship between music, the body, and movement accompanies us from the moment we are born and throughout our lives; babies and their parents use musical gestures that allow them to exchange experiences and emotions [28]. While sounds evoke a tangible initial physical movement in various organs of the auditory system, they also have a physical impact on the entire human body: the muscles, circulatory system, and nervous system. In fact, vibrations caused by sound waves affect every single atom in our bodies. In this sense, the human body can be seen as a “sound box” [29].

Musicking is the person’s ability to experience being part of a musical performance, which may include listening as well as producing music [30]. Bonde [31] proposed the term “health musicking” to note the power of music to promote health and wellbeing in the daily lives of each person, defining it as: “a corrective or affirmative emotional and/or relational experience created or facilitated by musicking” (p.122). Musicking can be experienced individually, with another person or in a group. The meaning of musicking lies in the relationships created between the individuals taking part [30]. When playing music in a group, individuals are able to express themselves, listen to themselves, and listen to others simultaneously, which leads to a sense of belongingness to the group without necessitating eye contact [32]. The musical harmony creates a motor synchronization and a sense of “we-ness” [33].

### The present research

The idea for the present research emerged from the professional staff of the music conservatory who are leading this project. We (the researchers) responded to this unique opportunity to learn and examine the subjective phenomena of musicking among people who suffer from PTSD. Since the treatment of PTSD is a challenge worldwide, we were also hoping to deepen our understanding about the therapeutic role of “health musicking”.

#### Authors’ statements

**First author:** My affinity to this specific subject matter has two aspects. First, the experience of musicking has been familiar to me since I was young, and I sing and play music as a hobby. Second, in my work as a psychologist, I am interested and have experience in treating PTSD. My clinical worldview is integrative, I integrate dynamic, CBT as well as DBT techniques. **Second author**: My clinical worldview is based on dynamic theories but it also includes nonverbal modalities such as music and movement. Music plays a central role in my life.

## Materials and methods

### Participants

Ten men aged between 31–60 and all diagnosed with war-induced PTSD participated in this study. All participants were veterans diagnosed with PTSD and treated in a psychiatric unit of a civil hospital in Israel. All participants took part in the music program organized by a music conservatory in a city in central Israel, which included weekly individual music lessons and a monthly musical enrichment group. The weekly individual lessons were frontal and lasted one hour. Each participant was matched to a teacher who taught him to play a chosen instrument. The monthly group meetings each lasted for two hours and were directed by various professional musicians, aiming to enrich the musical knowledge and curiosity of the participants. The program’s staff included a psychologist, drama therapist, volunteers, and music teachers who are not therapists. We conducted interviews with 10 of the participants, although there were 15 participants in the program at the time, during the second year of the program. They had participated in the program for anywhere from two months to two years and three months at the time of the interview. Their chosen instruments were guitar (3), piano (3), drums (3), and flute (1). All the interviewees are native speakers of Hebrew (see Table 1).

**Table 1:** Participants’ details.

Name (pseudonym)	Previous years of experience in music playing	Months in the program	Musical instrument
Yotam	3	8	flute
Assaf	23	20	drums
Shlomo	2	24	piano
Eithan	2	2	guitar
Amir	2	19	drums
Arye	5	24	piano
Omer	15	10	guitar
David	2	2	piano
Moshe	25	12	guitar
Haim	7	2	drums

### Ethics

This study was approved and conducted in accordance with the ethical guidelines of the ethics committee of the Academic College of Tel Aviv-Yaffo, Israel (number: 2018076). All participants signed a written informed consent according to the Helsinki Declaration and participated in the research voluntarily. The interviews were all coordinated with the staff of the music program and the data was analyzed anonymously.

### Research method

Qualitative analysis was chosen as the appropriate method for the present research. Several methods of qualitative analysis were considered before deciding on the interpretative phenomenological analysis (IPA). IPA aims to convey the participant’s own perception of an experience rather than present an objective account of it. For this reason as well as the narrative nature of its final analysis and its usefulness in portraying complexity and novelty, IPA was chosen for this study, enabling the creation of an analysis that goes beyond the standard thematic one [34]. Accordingly, a semi-structured qualitative interview was conducted, recorded, and transcribed, following Smith and Osborn’s [34] guidelines. Semi-structured interviews enables the interviewer to get close to the inner world of the interviewee, while accommodating the interviewee’s preferred pace and degree of exposure. A set of questions was prepared by the first author, who conducted the interviews. The two authors discussed the questions and then asked an experienced qualitative researcher to review them until they reached an agreement on which questions to present.

The interview began with a general, open question (“Please tell me what music is for you”), which each interviewee answered without interruption or interference. This was followed by additional questions regarding their experiences in the music project (“Please tell me about your experience with the program,” “Are you willing to share with me your experiences when playing?” “Does this experience stay with you after the meeting?”), which aimed at ensuring that the collected information was relevant to the study. Gentle prompts were used as the interviewer tried to complete pieces of the timeline or when he sensed that participants needed encouragement to provide more details or to speak more freely.

### Procedure

The music project is a collaboration between the psychiatric department of one of the hospitals in the center of Israel and the music conservatory. Participants were introduced to the project by the medical and paramedical staff of the psychiatric department, with participation based on self-referral. Participants pay a nominal fee for taking part in the project. The project manager matches between participants and teachers. After obtaining the consent of the music conservatory and the approval of the ethics committee of the Academic College of Tel Aviv-Yaffo, the first author began taking part in the monthly meetings of the music project, and participants were informed of his position. After four months the author began approaching participants, asking if they would be interested in being interviewed for the study, and scheduling a convenient time and place with those who agreed to be interviewed. Before the interview, participants were asked to sign an informed consent form with all the relevant details. The interviews were conducted individually, lasted between 30 and 60 minutes, and were recorded with the participant’s agreement. Participants were able to stop the interview whenever they wanted and were free not to respond to any questions that made them uncomfortable. Two interviews were not recorded due to the participants’ discomfort at being recorded. In these cases, the main points were jotted down during the interview and were transcribed immediately afterwards by the interviewer based on his notes and his memory. Participants’ personal details were concealed in order to protect their privacy.

### Data analysis

All interviews were transcribed by the interviewer and then analyzed by him and another researcher based on the four stages recommended by Smith and Osborn [34]. In the first stage, phrases were extracted from each interview and grouped into categories. The second stage comprised a comprehensive analysis of the categories designed to identify key themes. The researchers performed this stage separately and independently and then, together, chose the themes that accurately represented the interviewees’ experiences. In the third stage, a closer examination of the data led to a more abstract description, revealing three superordinate themes. The analysis was completed in the fourth stage with a higher level integrative narrative complemented by an intensive discussion of the findings and possible interpretation based on a comprehensive theoretical basis.

Several strategies were used to strengthen the plausibility of the results. First, consultation with another qualitative researcher offered an additional perspective on the data throughout all phases of the study. Second, the circular manner of the analysis forced the authors to consistently check for coherence between their interpretations and the narrative. Third, direct quotes are presented below to allow readers to explore the links between the data and the conclusions [35].

## Results

The data collected in the interviews led to the identification of two central themes: musicking as an intra-subjective experience and musicking as mediator of inter-subjective relationships.

### Musicking as an intra-subjective experience

This theme focuses on the subjective way in which musicking is experienced (including physical sensations, emotions, and thoughts) and becomes a part of the interviewees’ identity. It includes four categories: 1. music and instrument playing as a nonverbal emotional language; 2. musicking as evoking traumatic content and PTSD symptoms; 3. music and instrument playing as a protected space connected to childhood memories; and 4. musicking as a significant and central component in the development of identity.

#### Music as a nonverbal emotional language

*Music for me is simply another language to speak in*. *I love languages*, *I speak three different languages…music is another one and also the most beautiful of all*. (Yotam).

Most of the interviewees described music and playing a musical instrument as pertaining to their emotional world, using words such as “spirit” and “soul.” We identified two specific ways in which musicking was found to connect to the interviewees’ emotional world. First, the act of listening to music was described as an external emotional element that penetrates their internal world. Comparison was made a few times between musicking (both listening and playing) and taking medication, smoking cannabis, or experiencing relaxation.

*It’s hard to explain*, *it takes me apart*, *splits me*, *in a good way…You listen to a song and you feel as if your soul is tearful*, *you are not crying but your soul is*. (Amir)

Second, playing music was described as enabling the expression of emotions:

*I don't play well, but suddenly when we start, it’s…wow!… .Playing is ultimately connected directly to the soul*. *I only know two or three chords, but at the end you take it from inside and out of you in a way that puts you in touch with the emotion*. *(Eithan)*

#### Musicking as evoking traumatic content and PTSD symptoms

A number of interviewees reported that while playing they experience symptoms of emotional and physical hyperarousal as well as invasive thoughts and flashbacks. The encounter with the traumatic content is experienced as both positive and negative, which is why they sometimes find themselves avoiding this encounter yet at other times are able to cope or continue playing. For Shlomo, musicking allows him to encounter traumatic content without having to take action to avoid it:

*I recall people, events, smells, many relevant things*…*the memories evoked while playing [music] allow me to live in peace with them, thanks to the music*.

However, some interviewees reported being flooded with negative thoughts while playing as well as emotional and physical hyperarousal, thus making it a complex experience. For example, Amir reported:

*After 25–30 minutes of playing, usually things begin to get complicated for me, and I again start with the thoughts that “I am a shadow of myself,” that “I missed the boat”…as if even when the feeling is good*, *there is always something that slowly becomes stronger until my mind wanders, and the playing is unstable, and so on*.

When hyperarousal while playing a musical instrument is experienced as overpowering, different coping methods are used such as taking sedatives, smoking cannabis, taking a break from playing music, or engaging in physical activity.

Interviewer: *So how do you actually overcome it [the hyperarousal/anxiety attack]*?

Assaf: *I take pills*, *Clonex*, *Cipralex… I cope*, *or I hit myself to make the pain stop*, *or I take a pill to calm down*.

Playing music entails coping with difficulty, which is often experienced as failure but at other times as success, leading to a sense of satisfaction, enjoyment, and self-efficacy. Interviewees described how failure and success as part of the process of learning to play music become intertwined in their day-to-day need to cope with the rigidity and the self-criticism that are characteristic of PTSD.

*The hard part is a bit of stress*, *you want to do everything right*, *and then because I am a type of ah…you know*, *I hate to be wrong… so when I’m wrong I get into this anxiety*. *But I am slowly learning*. *When I do it and I see that it gets better and see what I succeed in doing*, *then its good*, *it becomes more fun*. (David)

The autonomic hyperarousal experienced in PTSD is also expressed somatically. Interestingly, among a number of interviewees we noted a concordance between the physical symptoms that they experience and the musical instrument that they chose to play, whether consciously or unconsciously. For instance, Yotam, whose responses are expressed mainly in chest pressure and pain, chose to play the flute, which requires use of the respiratory system:

*For me the physical pain here in the chest* [points toward his chest] *is very strong*, *so there is always the fear that breathing and extracting air will trigger [the pain]… I say metaphorically*, *that my flute has become the “oxygen tube” for my life at the moment*.

Arie, whose hands become rigid during anxiety attacks, chose to play the piano:

*I can’t move my hands, it’s also something to do with the post-trauma…something… it’s as if I cannot move my hands, they don’t obey me*. *As if I see the notes and the hands don’t move…The post-trauma caused my hands to be rigid, and I simply wasn’t able to play with my hands*.

Another interviewee, Assaf, who suffers from tension in his hands and needs to physically unload this tension chose to play the drums.

We can cautiously conclude that the process of learning to play an instrument is linked to the process of coping with PTSD. The act of music playing allows for emotional expression while also evoking positive and negative experiences. As it is a controlled activity, success in playing a musical instrument may lead to a sense of self-efficacy, satisfaction, excitement, vivacity, relaxation, and emotional expression. However, the emotional encounter also entails coping with symptoms of hyperarousal, physical and emotional pain, and the experience of failure that are all characteristic of PTSD.

#### Musicking as connected to childhood memories

All of the interviewees reported listening and being exposed and attracted to music from a young age. Their dealing with music in the present was thus a return to childhood memories.

*I started to play again at 32…I originally started as a kid and I remember it… because when I was already coping with my crises with post-trauma I escaped to all sorts of childhood memories that… I keep with me… I remembered how it is to begin “fresh” as a kid*, *especially when I began playing the drums again at 32*. (Assaf)

*My father used to listen to Pink Floyd with me at home…When I went to a Roger Waters concert in 2006*, *I think*, *I had a very intense emotional moment with tears which threw me back into childhood*. *I called my father and told him*, *“I wish you were here with me…now*.*” Just like the title in one of their songs*. (Eithan)

For others, acknowledgment of their love of music and their wish to play were always in the background but not realized. Playing at an older age after a number of years of coping with PTSD was, for these people, the fulfilment of a childhood dream.

*When I was a child*, *whenever I would see people playing the piano*, *it had a sort of “glory” in it*, *as if they play and everyone watches and they move their heads this way and that and it simply attracted me*. *I said to myself*, *“that’s exactly you; this is how you can express yourself emotionally*.*” But I never took it any further*. (Shlomo)

The act of playing an instrument was described by a number of interviewees as experiencing a game in a protected and isolated space that gives them the sense of security they recall from childhood.

*This is my kingdom [the drum set]*. *It’s like…when we were kids building a tent from pillows and a blanket and imagining we are in a spaceship or a plane or whatever… I used to disconnect from reality and be in my happy place*. (Assaf)

#### Musicking as a central component in the reconstruction of identity

Interviewees described musicking as a central component of their current identity. For some of them playing their instrument had become a significant, daily occupation.

*I play every day…the flute has really become the main thing in my life at the moment in terms of interest*, *I feel I am doing something*. (Yotam)

*It's the only thing I can concentrate on and stick with* … *it's the only thing I can focus on for half an hour*. *There were times that I couldn’t do even this* …*at first I managed to play a few minutes*, *and slowly*, *slowly more and more*, *it was like sparks that became a little light over time*. (Amir)

Some even described music as a basic need:

*Music*, *it’s almost like a need*, *I would say biological*, *like food or sex*, *something very basic and actually biological*. (Moshe)

However, for others, music was something they experienced only during the lessons.

*It is totally clear to me that I am completely into this thing [playing]… I feel it simply holds me*, *I hope I am not being too dramatic…for over the year now that I have been learning*, *I have not purchased a piano at home*, *so I only play the piano once a week…I look forward to my lesson once a week*. (Shlomo)

There is clearly a substantial variation between interviewees in the amount of time invested in musicking both in terms of the number of days a week they play and the duration of each session. For Amir, half an hour of playing is enough, while Yotam plays and practices for a few hours every day and Shlomo plays once a week during his lesson. Nonetheless, all three can be seen to experience their musicking as a significant and central component in their lives. It should be noted, however, that the adoption of musicking as a significant component of their identity is not derived from the objective amount of time and resources invested in it; musicking is adopted as part of the interviewees’ identity according to their subjective experience of themselves as active in a certain scope, framework, and mode that suits them individually.

Some of the interviewees demonstrated an aspiration to continue doing music in future.

*I want to be a master*, *I want to teach*. (Assaf)

*I am not a musician yet*, *but I can say that I am learning*. (Yotam)

This first central theme of musicking as an intra-subjective experience thus demonstrates the ways in which musicking–the listening, playing, and learning of music–forms a significant component of the interviewees’ emotional experience, which includes an encounter with both the various difficulties evoked by PTSD and positive emotional experiences such as satisfaction, motivation, self-efficacy, and vivacity. Moreover, we identified a pattern that presents musicking’s contribution to the development of identity by linking the interviewees’ current experiences to childhood.

### Musicking as mediator of an inter-subjective experience

Musicking was presented by the interviewees as a mediator of interpersonal relationships in two encounters with the outside world: the interaction with a single person, such as the teacher or a staff member, and relationships between the individual and his social environment.

#### Musicking as mediator between two individuals

*Relationship with the teacher*. Interviewees noted that during lessons they mostly play in the presence of the teacher, sometimes playing alone and sometimes playing together. The experience of playing with the teacher was described by a number of interviewees as causing insecurity, anxiety, and difficulty in concentrating.

*It’s even more stressful*, *but it passes and it works*, *it’s good*, *but it also comes with extra anxiety because it’s something that is added to playing because you have to listen*, *play together with her [the teacher] and keep up*. *But again*, *these anxieties…you know*, *sadly*, *are silly*, *not something that a normal person thinks about on a daily basis*. (Yotam)

The implications of exposing one’s weakness was often manifested in the appearance of PTSD symptoms during the lessons. However, positive feedback from the teacher was described as strengthening the motivation to persevere.

*Each time I do something he [the teacher] is so enthusiastic*, *it’s as if he’s happier than I am that I am succeeding*, *so that’s really fun*. *Working with him is really fun*, *making me want to come and continue doing it*. (David)

A number of interviewees described their teacher as someone they admired and followed:

*I admire him*. *The advice he gives me*, *I admire it*. *When I come to him agitated*, *broken*, *and depressed*, *he gives me the right answers*. (Assaf)

Some even perceive the teacher as a therapeutic figure:

*It’s like another psychologist for me…*.*You need to be a person who has experienced emotional upheavals…I can say he is a “music therapist*.*”* (Eithan)

The interpersonal space in which the lesson takes place is free of judgment and allows for the expression of difficulty and weakness. With time, the sense of calm and security in the interpersonal space seems to grow stronger, allowing the interviewees to feel comfortable about making mistakes, which in turn contributes to their collaboration and perseverance.

*Relationship with the staff*. Most interviewees mentioned at least one staff member with whom they had established an interpersonal relationship that motivated them to persist.

*You need a certain push*, *and they come and give it to you*, *letting you see that it’s even possible*: *“come and see and try*, *and if you don’t like it choose another instrument*.*”* (Yotam)

A few interviewees mentioned that, at the beginning, they had been wary of the staff, their motives, and their intentions but that, with time, this circumspection was replaced by trust and gratitude.

*This whole concept*, *I didn’t understand it at first and I thought*, *why is she calling me all the time*? *Coming to my classes*? …*What’s the catch*? *Most of the guys in the project kept thinking*, *there’s something strange here*, *maybe we are being taped*, *experimented on*. *We were all a bit apprehensive… but that’s the way it is*. *These people here*, *all they want is to give*. (Eithan)

It can thus be seen that the interpersonal ties between interviewees and the program’s teachers and staff that are an integral part of musicking began with the interviewees being wary and insecure. However, as they persisted with musicking, these relationships are increasingly experienced as an added-value interpersonal emotional space. Often the teacher was described as a soulmate, a therapist, and an object of identification and admiration, and relationships with members of staff also served a motivational role contributing to perseverance. The study of music and the relationships derived are experienced as intertwined, and musicking therefore forms a significant part of the interpersonal relationship, extending well beyond the social encounter.

*I am lucky*. *I was blessed by God to have such a person enter my life and constantly organize my chaos*. *Even beyond the actual drums*, *he [the teacher] helps me get things that I experience into perspective*. *Even my drum playing he manages to put in perspective*. (Assaf)

#### Musicking as mediator between the individual and society

Besides the personal relationships, we found musicking to have a meaningful role in the connections between the interviewees and their social and cultural environments. This category is comprised of two different aspects: the first is a yearning for relationships yet difficulty in establishing social bonds, and the second is the musicking space and environment as a social and community circle that is free of social stigma.

*Yearning for relationships* Most interviewees mentioned the group meetings as a place where they are exposed to their struggle to establish ties with other people. However, despite this, we found among them a yearning for a sense of belonging and for the project’s group meetings to become a meaningful social environment.

*It was a bit surprising for me to see that most people don’t communicate with each other*. *I would have liked there to be more communication…it would be nice to talk a bit more with each other*, *and not only when we come and go*. (Yotam)

Some interviewees expressed this yearning in their wish for a social musical activity that would enable the creation of social bonds.

*I think that if there were more social events*, *going to some musical thing… we were once taken to a show at a bar*, *it was really good*, *I really enjoyed it*. *I miss going out and being in something social like that*. (Amir)

Similarly, a few of the interviewees noted that they would like, at some point, to play music together with other people.

*You know*, *after each of us has practiced in his room with his teacher*, *there is a meeting where everyone is together*, *and there was even one meeting when we played and it was incredible*. *Each one in separate rooms and then suddenly we come and try to create something together…it’s fun*. (Eithan)

Interviewees who have experienced playing music together with others elsewhere described it as addressing the need to connect with others.

*Playing a musical instrument is like another language that you learn to speak and then you can speak to people who speak that language*. *The ability to play*, *express yourself*, *speak with others with music is something that cannot be taken for granted*. (Omer)

Overall we found that the interviewees aspired to establish social ties in their group meetings while at the same time experiencing difficulties. The experience of musicking may address their need to connect with others. It is important to note that among a number of interviewees who have another musical social environment that addresses this need, the current project is experienced as a less significant social environment and they displayed less of a yearning to connect with others in this project.

*Musicking space as a social framework free of social stigma*. In general, interviewees experience the program as a social framework that provides them with a sense of belonging because of the absence of social stigma. The issue of stigma was raised in the interviews in two contexts: the inter-group context, namely, the way each person perceives their identity in comparison to other participants and staff members, and the comparison to society in general. The program is experienced as a community framework for people dealing with a common issue, and this cancels out the difficulties provoked by the stigma of being “ill” or “post-traumatic.”

*In this environment you feel that you are with people who have been hurt just like you*, *and you don’t feel outside of society*. *In normal society*, *you do feel different*, *because of behaviors like getting up in the middle of a meeting*. *There are those who do this with their feet* [illustrates by stamping his feet], *people don’t have patience…here everyone knows this and understands*, *and even the staff*, *and it’s… like going to the pub with the guys*. *These are not [my] best friends*, *but it still feels like a familiar place*. *(Arie)*

The physical environment of the music conservatory was also described as different from a hospital.

*The fact that we are sitting here [in the music* conservatory*] and not in a hospital gives a sense of something that is not “therapeutic” or “rehabilitative*,*” but something fun*, *a learning experience*, *something that can contribute a lot to each of us in his caring and his love for music*. (Shlomo)

*The thing I most dislike is going to hospitals*. *That’s where I feel the most fucked up*. *That’s also why I wouldn’t go to a rehabilitation ward*. *I don’t want to go there*, *because if you are there*, *that’s it*. *You are screwed*. *Here [at the music* conservatory*] you feel that you are coming for something that makes you feel good*. (Amir)

The physical space of the music conservatory is also experienced as one that creates a relaxed atmosphere of enjoyment and domesticity.

*There is something in this place that makes me like coming here*, *even though I don’t know what…the garden…the building*, *I like coming here*, *so if I have a reason I come*, *if it’s a concert*, *a program meeting*, *or a lesson…I don’t know*, *it just makes me feel good*. *I come here for four hours and smoke only one joint*, *while usually I smoke much more to balance myself*. *In other places I can smoke seven joints in four hours*. (Amir)

The space is regarded as free of social stigma and characterized by a sense of belonging and recognition by society and community. Interviewees described group encounters with well-known musicians whom they admire as expanding their horizons and giving them a sense of social recognition.

*We have people coming here who know how to play the violin and teach us and…this whole brainstorming is something wonderful and certainly contributes to us especially enjoying these sessions*. (Shlomo)

*The greatest artists came without ego*, *without pretention*, *without anything*. *They came for us*, *to identify with us*, *to strengthen us*, *and it’s amazing*. (Eithan)

A number of interviewees noted that beyond the music lessons and group meetings they also attend concerts at the music conservatory that are open to the general public.

*I try to come to everything*, *I also go to all the concerts taking place here at the music conservatory*. *We get free tickets*. (Yotam)

The project thus creates a sense of belonging to a community that is united by its members’ musicking. Some interviewees even felt comfortable and secure enough to take part in wider community musical activities beyond the scope of the program.

To sum up these results, musicking was found to serve as a space free of social stigma that allows for coping with the yearning for interpersonal ties while struggling to establish them. As the musicking is not directed toward therapy nor is it socially defined or perceived as therapy, project participants are able to experience themselves as having a social and communal common denominator that is not derived from their medical or emotional condition. This perception is further enhanced by the fact that the musicking takes place in a physical space that is not “therapeutic” or “rehabilitative” but is rather a social and cultural institution intended for the well-being of the public at large. All of these factors create a human and physical environment in which participants take part in different circles of social and community musicking without feeling different or experiencing social stigma.

**Fig 1:**
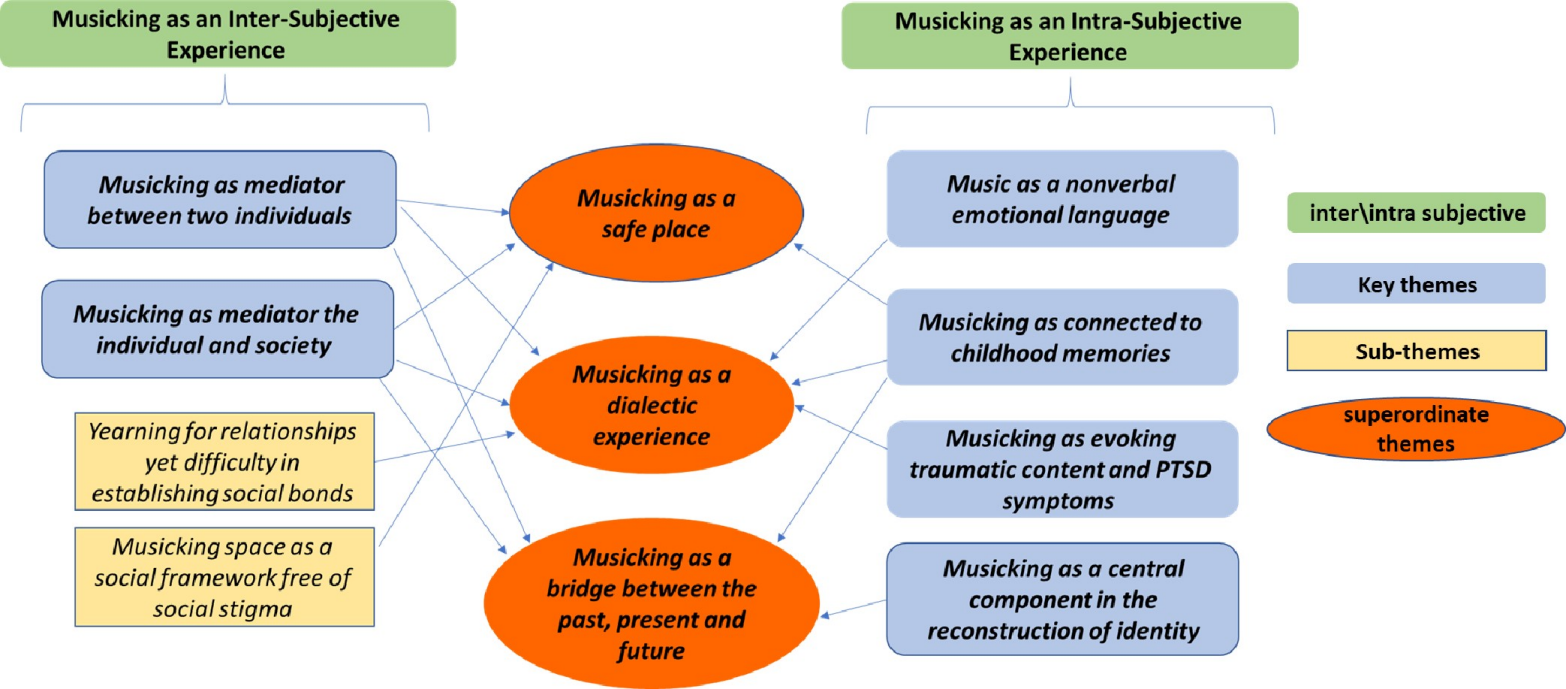
Relationships between themes.

A closer examination of the data allowed for the creation of three superordinate themes that represent the whole process: 1.musicking as a safe place; 2.musicking as a dialectic experience; and 3. musicking as a bridge between past, present and future. These three superordinate themes connect a narrative in which musicking creates a safe space for identifying the self and reconstructing a coherent personal narrative while performing a dialectic movement between pain that is connected to traumatic experiences and a sense of vitality. The relationship between the themes is presented in Fig 1.

## Discussion

The discussion on the findings will incorporate the three superordinate themes and the narrative derived from them.

### Musicking as a safe place

We approach the theme of musicking as a safe place from a psychoanalytic point of view, following some of Winnicott’s theoretical conceptualizations that include play, transitional phenomenon, and holding [36]. Accordingly, children “search for the self” in the potential space between internal and external reality where they feel safe and unthreatened [36]. This process is achieved through play, defined by Winnicott as a transitional phenomenon.

Trauma resulting from war has often been described as a stressful event that causes regression characterized by a sense of dependence and neediness [37]. Parson [38] argued that following exposure to the trauma of war, individuals experience a threat to their self-cohesion. Symptoms such as anxiety, apathy, and emotional detachment, inability to grieve, damaged self-esteem, and difficulty in emotional regulation often followed by uncontrollable rage are examples of the ways in which the fragmented self is expressed outwardly.

Based on the assumption that the post-traumatic individual is in a regressive state characterized by a damaged self-cohesion, we suggest a point of view which sees our interviewees’ spontaneous music playing as “play” taking place in a safe environment and thus allowing the post-traumatic individual to reconstruct their injured self, similar to children’s play.

Winnicott [39] argued that an important process in children’s development is the way in which they learn to play with others. At first the baby plays alone in the presence of their mother, with the mother serving as a mirror to the baby’s experiences. Gradually, the baby learns to play alone, separating from the mother, and finally learns to play together with the mother, seeing her as a separate entity. Considering the findings of the present study, music played in the presence of the teacher can be compared to this process: playing in the presence of the teacher, moments of playing alone, and finally playing together with the teacher. These active experiences contribute to the reconstruction of the wounded self.

Winnicott [40] coined the term “holding” to describe the mother’s adjustment to the baby’s rhythms in sleep and breastfeeding at the earliest stage of development. In the following stages, when object relations begin to evolve, the meaning of holding becomes “a psychological place where the infant may gather himself together” [41 p.1352]. Thus, the mother holds the baby’s entire experience of existence, both physical and emotional, which Winnicott called “primary maternal preoccupation” [42]. In a therapist–patient relationship it is the therapist’s ability to “hold” patients without suggesting verbal interpretations that provide them with a “human place” where they can experience themselves as a whole [41]. In the present study, this concept of holding within the teacher–student or student–staff member relationship can be identified in the interviews:

*During the lesson itself it’s as if the teacher already knows whom she is dealing with*, *she understood this very quickly*. *There is no lesson without an attack at one point or other*. *So I simply tell her to stop*, *I set the instrument aside*, *go out*, *take out the strong part of the pain*, *and then I come back inside all folded up*, *and she knows how to work with me on theory or to play something just to try and get through the last 15 minutes so as not to waste it entirely*. (Yotam)

*I feel that she simply understands me*, *I don’t know if it’s because she has been in this field for many years*, *but I feel she knows exactly when to insist and when to let go with me*. *She is truly a special woman*, *and I am in contact with her all the time*. (Amir)

The holding metaphor was applied by Applegate [43] to the macro level of the “holding environment” concept. “Holding environment” refers to a context in which interpersonal relationships exist and the manner in which vulnerable and weakened populations can be given an opportunity to express their pain and to experience themselves in cultural and social activity. Thus, within the relationships between the individual and a “holding environment” there is a potential space, which is experienced as a safe place.

*I like coming here…I don’t know*, *it just makes me feel good*. *I was even happy about coming an hour earlier today to meet you because it gave me another reason to be here [at the music conservatory]*. *I’m calm here*, *I don’t know why*. (Amir)

In addition, the actual music playing space, the music conservatory, experienced as a safe, unthreatening place and free of social stigma, can be defined as a “potential space” [40] in which individuals can get in touch with their authentic self. The experience of visibility and recognition by the music conservatory allows interviewees to experience themselves as “doing music,” i.e., musicking. This acknowledgment helps them to internalize the musicking and make it a part of their identity, in contrast to the usual social branding as “ill” and “post-traumatic” that they experience in medical settings.

*We don’t come to a psychiatric ward but to the music* conservatory. *It’s a sense that we came to play music*, *so yes*, *it’s something in the head*, *its different*, *you arrive*, *you come with an instrument*, *you are not coming to take your pills or something*, *and I think it’s an incredible idea that they did it there* (Eithan).

Winnicott’s developmental theory thus enables a conceptualization of musicking as playing, as a transitional phenomenon, and as taking place in a “holding environment” which enables a potential space while searching for the self. Musicking at the conservatory facilitates an expansion of the intrapersonal potential space into the interpersonal dimension. The project and the school form a safe place and are not experienced as part of the external world, which is predominantly felt to be a hostile place among individuals coping with PTSD. This interpersonal space nonetheless encourages dialogue, acknowledgment, and visibility by the external world while mediating between the interpersonal and the external world.

### Musicking as a dialectic experience

Following the two central themes, interviewees described musicking as a positive experience that also includes an encounter with difficulties. At the intrapersonal level, they described situations in which musicking leads to hyperarousal, which is one of the symptoms of PTSD, while at the same time they experience a sense of vitality and vivaciousness. Similarly, we identified situations in which the yearning for connection and harmony are experienced alongside insecurity, low self-esteem, social anxiety, and difficulty in making social contact.

The discussion about this superordinate theme relies on dialectic theory as well as current knowledge from other CBT theories and the field of psychophysiology. The dialectic theory views human experiences as comprised of opposed processes (thesis–anti thesis), which together create synthesis. According to this theory, a conscious state in which individuals live their lives with a sense of meaning is characterized by a constant transition between two modes: direct experience and explanation [44]. Dialectic behavior therapy (DBT) was developed according to this theory with the aim of treating people with borderline personality disorder. The theory assumes that emotional dysregulation is a result of both biological predisposition and an “invalidating developmental environment” which is characterized by abuse and a lack of empathy [45]. DBT principles based on this theory were later integrated in the treatment of PTSD [46]. DBT has three main purposes: 1. attaining emotional regulation by acquiring behavioral and cognitive techniques alongside an emphatic and non-judgmental approach by the therapist; 2. coping with symptoms via exposure therapy in order to experience the threatening stimuli in a safe environment; and 3. developing meaning in one’s life through a verbal discourse that grants symbolic meaning to the experience [47]. Based on our findings, musicking addresses the first two purposes of DBT, while the third purpose is achieved via its nonverbal characteristics.

Rothschild’s [7–8] theory can be adopted to explain the dialectic nature of the interviewees’ experiences. While, on the one hand, the arousal of the amygdala by musical stimuli might evoke the physical memory of the traumatic events, on the other hand, findings have shown that playing a musical instrument and listening to pleasant music facilitates emotional regulation by increasing the efficiency of the frontal cortex and reducing activity in the amygdala [48]. For instance, a meta-analysis conducted by Moore [48] found that participants described both negative emotions while playing as well as a positive emotional component linked to the musicking experience. Likewise, in the current study, interviewees noted that often while playing their musical instruments they were able to bear the hyperarousal and invasive symptoms. One possible explanation is the “flow” experience of playing a musical instrument, which forms a motivational factor for persistence and satisfaction [49]. A “flow” experience is an action that requires high concentration, balancing between the demands of the task and the person’s capabilities. This action is experienced as automatic and conscious, including a sense of control and characterized by lack of self-criticism. The purpose of this action is the act itself, which holds sufficient value and satisfaction [50]. The findings of the present study correspond with the findings of Bensimon [32] who found that during group drumming, people coping with PTSD experienced pervasiveness and emotional hyperarousal symptoms, but it allowed them to get used to the sounds and the musical stimuli that evoked these symptoms.

Interviewees in the present study described music as a nonverbal emotional language, expressed by playing a musical instrument; hence, while playing, they also experienced the construction of meaning. Stern’s analogy theory [51] sees musical expressions as analogous to “vitality affects.” Accordingly, the initial communication between a parent and child is nonverbal. Babies, who cannot speak, express their internal world (“vitality affects”) by voice and movement in order to engage their parent’s attention. The structure of this behavior is identical to the cornerstones of music including intervals, volume, compression, pitch, rhythm, and more. Thus, the basic concept in music therapy is that patients’ musical experiences are analogous to their intra-psychic processes. Another concept relates to the idea that meaning is interwoven in the musical form itself. There is therefore no need for verbal or cognitive interpretation of the musical experience in order to give it meaning [52]. Using this analogy theory, we can conclude that in the present study musicking allows interviewees to express “vitality affects” and experience their internal world as meaningful as they nonverbally process it.

We therefore use DBT terms to suggest that musicking is experienced as a dialectic process. The encounter with the traumatic memories and their symptoms (exposure) goes hand in hand with positive experiences involving a sense of vitality, satisfaction, enjoyment, emotional regulation and developing meaning which enables self-development. Musicking holds, on the one hand, a yearning for social contact and harmony, and on the other hand, involves suspicion, anxiety from intimacy, and fear from the exposure of weakness. In the encounter examined here, the outside world is represented by the teacher and other group members. The social framework in which the participants feel normal can be seen as a “validating environment” (the opposite of an “invalidating environment” [45]), which is sensitive to their situation and contributes to their emotional regulation. Among interviewees who have experienced playing their instrument together with others, the experience was described as one that provides a sense of nonverbal connection and communication with others and a means of regulating their social anxiety.

As mentioned above, physical concordance was often found between the somatic symptoms experienced by the interviewees as part of their PTSD symptoms and the musical instrument that they chose to play. Hence, the emotional expression in musicking also includes a physical aspect which is expressed in the individual’s choice of instrument. A possible explanation integrates Rothschild’s [8] definition of “implicit memory” with Stern’s analogy theory [51]. According to Rothschild [8], a traumatic memory is only available on the sensory and physical level. Thus, creating music, which involves sounds and motor skills without a verbal meaning and is therefore analogous to experiencing “vitality affects” [51], may serve as a means of symbolic processing and integration of the contents of the traumatic event. Within the context of this finding, playing music can be seen as a sublimatory process and the instrument as an object that addresses an urge that derives from the body and searches for release in physical areas where somatization takes place. Sechaud [53] views sublimation and somatization as two internal processes which can exist in parallel, as in a traumatic state when part of the self is dissociated. Sublimation and somatization are intertwined, forming a movement involved in coping with the trauma. From this point of view, playing music as sublimation among the interviewees may point to an internal movement directed at relinking, connecting, and reorganizing their internal worlds. Such an interpretation may be reinforced by the argument that musical instruments are a representation or expansion of the player’s internal experience and a way of enabling physical contact between individuals and the world around them [20].

It can thus be seen how each of the goals of DBT (emotional regulation, exposure, and developing meaning) are expressed in musicking among people coping with PTSD. We therefore recommend musicking as a way of creating a shift from a state of avoidance to a dialectic encounter that involves exposure to the negative PTSD symptoms alongside positive feelings of satisfaction, enjoyment, relaxation, and vitality.

### Musicking as a bridge between the past, present and future

This superordinate theme focuses on the way in which musicking is expressed as a substantial part of the reconstruction of identity. The discussion on this topic relies on narrative theory that views the individual’s identity and concept of self as a product of the life story they tell themselves; a coherent personal narrative thus represents a sense of completeness of identity. A basic assumption of this approach is that the narrative is a product of constructive cognitive action [54]. The construction of a personal narrative is based on cultural, experiential, and historical contexts [55]. Frank [56] coined the phrase “chaos narrative” which represents a story following a traumatic event and is, in fact, an anti-narrative. Rimmon-Kenan [57] argued that trauma causes disruption in the personal narrative sequence, undermining continuity and coherence. This results in a split in the identity between the period before the traumatic event and the time after it. She claimed that narrative reconstruction is an ongoing process, which may include segments of Frank’s [56] “chaos narrative” coexisting with coherent segments. This argument corresponds with Woolf’s [58] findings that the chaos narrative exists in parallel to other narratives among US veterans coping with PTSD.

The interviewees in the current study presented a narrative which related to the place of music and musicking in their lives. The narrative includes three stages pertaining to different periods in their life. The first period, childhood and youth, was described as a period of attraction to and love of music. The second period is young adulthood, during which musicking was not central in their lives. This period includes their military service, their traumatic experience, and the onset of PTSD. The third period is late adulthood, during which musicking has taken a central part in their lives alongside their coping with PTSD. Among some of the interviewees, musicking was described as a return to an activity that was central in their childhood. For others, musicking and, specifically, playing a musical instrument was described as the fulfillment of an identity component that had not yet been realized. The interviewees’ narratives can thus be characterized into two types: musicking as a renewed identity component and musicking as part of a new identity.

Rimmon-Kenan [57] emphasized the importance of continuity as a form of coherence. Continuity pertains to the time and chronological sequence between the past, present, and future, and she defined three ways in which the continuity of a narrative can be achieved: narrating the past in light of the present; narrating the present in light of the past; and realigning present and future. Musicking may, accordingly, serve to create continuity in the narrative of our interviewees. For those who played a musical instrument in their childhood and returned to playing at an older age, musicking may narrate the present in the light of the past (i.e., “I used to play and now I have gone back to it”). For those who embarked on musicking only later in life, musicking may narrate the past in light of the present (i.e., “today I am doing what I dreamed of doing in the past”). A realignment of the future is expressed in the fact that interviewees see themselves as involved in musicking in their future lives.

Identity and narrative are a complex system of personal experiences, interpersonal social relationships, and cultural attributes. The narrative can never be entirely personal, as we construct it in a specific cultural context and in the context of the existence of others around us [59]. The interviews demonstrate that musicking has introduced possibilities for establishing interpersonal bonds during the project and contributed to a renewed sense of belonging to a social framework as part of the construction of identity. It is important to note that for most interviewees, at the time of experiencing the traumatic event (the second period), musicking was not a central component of their identity. Hence, music and musicking are not experienced as characteristic of the chaotic and broken part of the personal narrative and are thus able, in the present, to bridge between coherent parts of the individual’s past childhood identity and their present narrative.

All of the above can be grouped into the following narrative: Musicking among PTSD interviewees is a “secure place” for their wounded self, allowing the reconstruction of a coherent personal narrative while conducting a dialectic encounter with the trauma and its symptoms using nonverbal language.

## Conclusion and recommendations

The present study provides a preliminary perspective of the subjective experience of musicking among men coping with war-induced PTSD. The findings of the study shed light on the ways in which musicking is integrated in interviewees’ internal experience. The diverse theoretical body used may reflect the various roles musicking takes in the experience of coping with PTSD and thus indicate the great therapeutic value of musicking. Processes involving emotional regulation, exposure, and construction of narrative that belong to behavioral and cognitive therapy methods were identified alongside processes of searching for the self while giving symbolic meaning that are ascribed to psychodynamic therapy methods. Despite the extensive and diverse therapeutic value identified in both the intrapersonal and interpersonal dimensions, musicking is experienced by interviewees as non-therapeutic in nature. Our conclusions thus support Bonde’s [31] claim that musicking has therapeutic and healing qualities even when conducted outside of a formal therapeutic setting. Consequently, we propose musicking as a therapeutic tool for people coping with war-induced PTSD from both the intrapersonal and the interpersonal perspectives.

## Research limitations and further research

The aim of the present study was to examine, from a phenomenological perspective, the experiences of 10 men coping with PTSD who took part in a project at a music conservatory. In view of the high sensitivity to exposure among this population, protecting the interviewees’ anonymity was essential for ensuring their collaboration and willingness to participate. For this reason, personal and demographic data, such as age, marital status, occupation, ethnic origin, religion, and the time of onset of PTSD symptoms, were not collected, although these may have shed further light on the findings. Moreover, two interviews were not recorded following the interviewees’ requests. These interviews were transcribed from the interviewer’s memory and notes taken during the interview, which may have impacted the findings.

Being a qualitative study, its conclusions rely on the interpretation of the researchers and the subjective experiences of the interviewees who agreed to participate. These conclusions are not objective, and therefore any generalizations based on the findings of the study are limited. Moreover, the study was based on a population for whom music is a passion. It is possible that for people who are not attracted to music, musicking would not be experienced in the same ways described in this study. It would be interesting to examine the experience of other creative activities for coping with PTSD. This may shed light on the uniqueness and different qualities of each form of creativity and explore the possibility of matching creative activity to individuals according to their character and areas of interest. We should further note that the interviews may have included an aspect of social desirability and that responses may be thus biased in view of the interviewees’ conscious or unconscious wish to be perceived positively by the program staff.

We recommend further longitudinal research that will enable observation of the process that participants are going through and their coping strategies and, in the case of a quantitative study, employ a few questionnaires.

## Reflections (first author)

Qualitative researchers are able to take the position of both insiders and outsiders of the field being explored [60]. My affinity to this specific subject matter has two aspects. First, the experience of musicking has been familiar to me since I was young, and I sing and play music as a hobby. Second, in my work as a psychologist, I am interested and have experience in treating PTSD. During the interviews I was introduced to various different stories that highlighted the extent to which the personal experience of coping with PTSD differs from one individual to another, likewise their experience of the role of musicking. Most people describe music and playing as a positive experience, which cannot be precisely described in words. For me, music is an experience to which I can devote myself that simultaneously calms my worries and daily thoughts. Through the experiences of the interviewees, the current study has acquainted me with other ways in which musicking takes part in my experience of music. Music is able to both influence the individual’s emotional state and evoke difficulties and severe pain. This aspect of musicking was, previously, less central to my personal point of view but has now led me to pay greater attention to moments in which musicking evokes difficulties for me as well. While writing this paper I have, in addition, thought extensively about the ways in which musicking has been part of the formation of my identity and personal narrative as a means of emotional regulation and providing a safe place.
